# Nonstationary Pharmacokinetics of Caspofungin in ICU Patients

**DOI:** 10.1128/AAC.00345-20

**Published:** 2020-08-20

**Authors:** Agnieszka Borsuk-De Moor, Justyna Sysiak-Sławecka, Elżbieta Rypulak, Michał Borys, Paweł Piwowarczyk, Grzegorz Raszewski, Dariusz Onichimowski, Mirosław Czuczwar, Paweł Wiczling

**Affiliations:** a Department of Biopharmaceutics and Pharmacodynamics, Medical University of Gdańsk, Gdańsk, Poland; b 2nd Department of Anaesthesiology and Intensive Therapy, Medical University of Lublin, Lublin, Poland; c Department of Physiopathology, Institute of Rural Health, Lublin, Poland; d Department of Anaesthesiology and Intensive Therapy, Faculty of Medicine, University of Warmia and Mazury, Olsztyn, Poland

**Keywords:** caspofungin, population pharmacokinetics

## Abstract

Standard dosing of caspofungin in critically ill patients has been reported to result in lower drug exposure, which can lead to subtherapeutic 24-h area under the curve to MIC (AUC_0–24_/MIC) ratios. The aim of the study was to investigate the population pharmacokinetics of caspofungin in a cohort of 30 intensive care unit patients with a suspected invasive fungal infection, with a large proportion of patients requiring extracorporeal therapies, including extracorporeal membrane oxygenation (ECMO) and continuous renal replacement therapy (CRRT). Caspofungin was administered as empirical 70 mg antifungal therapy administered intravenously (i.v.) on the first day and at 50 mg i.v. on the consecutive days once daily, and the concentrations were measured after three subsequent doses. Population pharmacokinetic data were analyzed by nonlinear mixed-effects modeling. The pharmacokinetics of caspofungin was described by two-compartment model. A particular drift of the individual clearance (CL) and the volume of distribution of the central compartment (*V*_1_) with time was discovered and described by including three separate typical values of CL and *V*_1_ in the final model. The typical CL values at days 1, 2, and 3 were 0.563 liters/h (6.7% relative standard error [6.7%RSE]), 0.737 liters/h (6.1%RSE), and 1.01 liters/h (9.1%RSE), respectively. The change in parameters with time was not explained by any of the recorded covariates. Increasing clearance with subsequent doses was associated with a clinically relevant decrease in caspofungin exposure (>20%). The use of ECMO, CRRT, albumin concentration, and other covariates did not significantly affect caspofungin pharmacokinetics. Additional pharmacokinetic studies are urgently required to assess the possible lack of acquiring steady-state and suboptimal concentrations of the drug in critically ill patients. (This study has been registered at ClinicalTrials.gov under identifier NCT03399032.)

## Abstract (precis)

Standard dosing of caspofungin in critically ill patients has been reported to result in lower drug exposure, which can lead to subtherapeutic 24-h area under the curve to MIC (AUC_0–24_/MIC) ratios. The aim of the study was to investigate the population pharmacokinetics of caspofungin in a cohort of 30 intensive care unit patients with a suspected invasive fungal infection, with a large proportion of patients requiring extracorporeal therapies, including extracorporeal membrane oxygenation (ECMO) and continuous renal replacement therapy (CRRT).

## TEXT

Caspofungin is an echinocandin antifungal agent widely used in treating invasive fungal infections in the intensive care unit (ICU) setting. Echinocandins are a safer alternative to more traditional polyene and azole therapies since they target cell wall absent in human cells and therefore cause fewer toxic events (1). Slowly emerging echinocandin resistance entails a need for more studies investigating relationships between drug exposure and patient characteristics, as well as for pharmacodynamic studies linking exposure with efficacy.

Critically ill patients have altered physiology and undergo multiple medical procedures, which can affect the pharmacokinetics of drugs. Standard dosing recommendations based on studies in healthy volunteers may not be suitable for special populations such as intensive care unit (ICU) patients (2). Studies in real-life clinical setting in critically ill patients are difficult, burdened with high variability due to multiple procedures performed in the ICU, drug coadministration, and patient instability, but are vital for the assessment of therapy in this group of patients (3).

Literature data on caspofungin pharmacokinetics in ICU patients are limited. Standard dosing of caspofungin in critically ill patients has been reported to result in lower drug exposure, which can lead to subtherapeutic 24-h area under the curve to MIC (AUC_0–24_/MIC) ratios (4, 5). The metabolism of caspofungin is mainly hepatic, so the patients with liver impairment require caspofungin dose reduction; the manufacturer recommends a 35-mg maintenance dose (versus a 50-mg standard dose) with a 50-mg loading dose in patients with moderate liver impairment, with no recommendations for patients with severe liver impairment (6). However, a study conducted by Martial et al. on adapted dosing regimens in patients with a Child-Pugh score B and hypoalbuminemia revealed that dose reduction leads to suboptimal exposure (7).

According to the drug label, dose adjustment is not required in patients with renal failure and requiring renal replacement therapy (6). This was confirmed by Weiler et al. in a study investigating the influence of various modalities of continuous renal replacement therapy (CRRT) on caspofungin exposure in critically ill patients (8). Nevertheless, Pérez-Pitarch et al. suggested that the licensed dosing regimens might be insufficient for critically ill patients on hemodiafiltration (5).

There are little data concerning the influence of extracorporeal membrane oxygenation (ECMO) on caspofungin pharmacokinetics (9, 10). ECMO is known to alter drug pharmacokinetics due to drug extraction during the procedure and a large volume of exogenous blood needed for ECMO circuit. The changes include increased volume of distribution and decreased clearance (11). The interactions between the ECMO circuit and the drug are not always easily predictable, but one of the vital drug characteristics determining circuit drug loss is the lipophilicity and protein binding (12, 13). Since caspofungin is highly protein bound (97%) and alterations in the levels of plasma proteins in critically ill patients are commonly occurring, the pharmacokinetics of caspofungin in ICU patients requiring ECMO therapy might be affected.

Here, we sought to investigate the population pharmacokinetics of caspofungin in a cohort of 30 ICU patients with a suspected invasive fungal infection, with a large proportion of patients requiring extracorporeal therapies, including ECMO and CRRT. We also explored the influence of patient characteristics and vital parameters on caspofungin pharmacokinetics and assessed drug exposure.

## RESULTS

**FIG 1:**
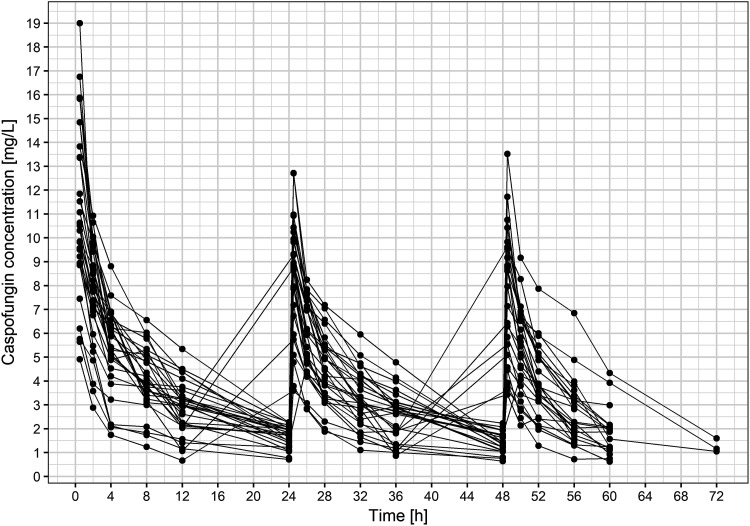
Caspofungin concentration-time profiles for all patients in the study.

**FIG 2:**
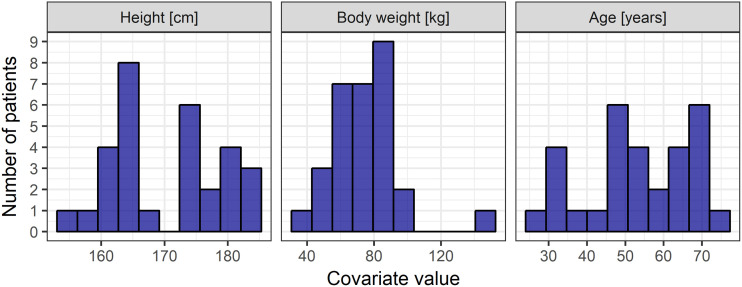
Distribution of height, weight, and age of patients in the analyzed population.

**FIG 3:**
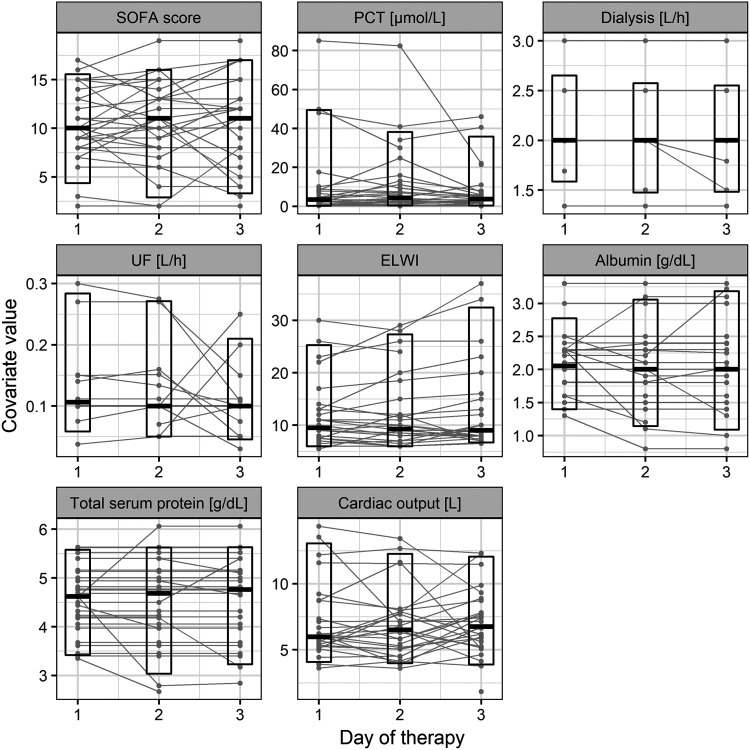
Summary of time-dependent covariates changing over days for the study population. The boxes cover the 5th to 95th percentile range, with a horizontal line denoting the median value.

The analyzed data consisted of 481 measurements of caspofungin concentration from 30 ICU patients. Six samples with concentrations below the limit of quantification were discarded from further analysis. Raw concentration-time data are presented in Fig. 1. The characteristics of the patients are presented in Table 1 and Fig. 2 and 3.

**TABLE 1:** Summary of the study population^*a*^

Parameter (unit)	Median (range), *n* = 30
Age (yr)	53 (28–76)
Wt (kg)	74 (40–150)
Ht (cm)	171 (156–185)
No. of subjects	
Male/female	16/14
CRRT (yes/no)	21/9
ECMO (yes/no)	10/20
Survival (yes/no)	13/17
Liver failure (yes/no)	3/27
SOFA score	11.0 (2.00–19.0)
PCT concn (μmol/liter)	3.72 (0.130–85.0)
Dialysis dose (liter/h)	2.00 (1.34–3.00)
Ultrafiltration rate (liter/h)	0.09 (0.03–0.30)
ELWI	9.00 (5.50–37.0)
Cardiac output (liter)	6.22 (1.84–14.4)
Albumin concn (g/dl)	2.00 (0.80–3.30)
Total serum protein concn (g/dl)	4.68 (2.67–6.06)

a Values are expressed as the median and range (except as indicated otherwise in column 1) for continuous covariates and as counted for categorical variables. CRRT, continuous renal replacement therapy; ECMO, extracorporeal membrane oxygenation; SOFA, sequential organ failure assessment score; PCT, procalcitonin; ELWI, extravascular lung water index.

A two-compartment model was chosen to describe the pharmacokinetics of caspofungin in clearance and volume terms. Interindividual variability was estimated for clearance (CL), volume of distribution of the central compartment (*V*_1_), and volume of distribution of the peripheral compartment (*V*_2_). The interindividual variability on intercompartmental clearance (*Q*) was not included in the model since the estimate was close to 0 in the model-building process. A correlation between CL and *V*_1_ was discovered in model diagnostics and included in the model since it significantly improved model fit.

**FIG 4:**
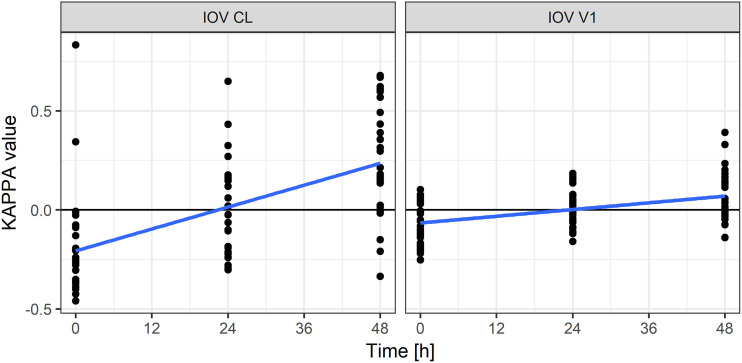
Relationship between individual estimates of kappa (deviation of the individual estimate from the population mean on each occasion) for clearance and central compartment volume of distribution and time. The blue line represents the linear trend in the data.

In the course of the modeling process, a model including interoccasion variability (IOV) was tested since the drug was given on three occasions. The CL-*V*_1_ correlation in this model was not estimated due to overparameterization. Since the model with IOV estimated for CL and *V*_1_ showed similar performance to the model with only interindividual variability (IIV), the estimation of IOV was abandoned. However, the model with IOV was utilized to investigate the relationship between individual pharmacokinetic parameters on each occasion of drug administration and time-varying covariates. The investigation showed no systematic relationship between parameters and covariates, but a particular drift of individual CL and *V*_1_ values with time was discovered (Fig. 4). This phenomenon was described by including three separate typical values of CL and *V*_1_ (different typical values for each occasion) in the IIV model.

**FIG 5:**
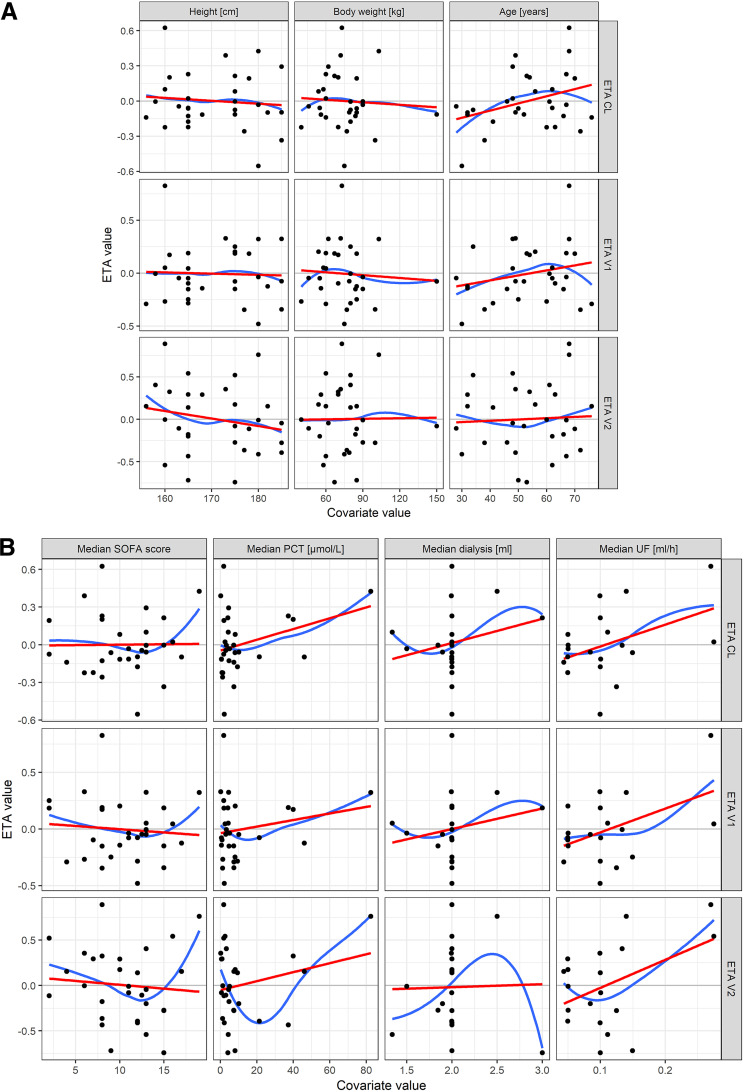

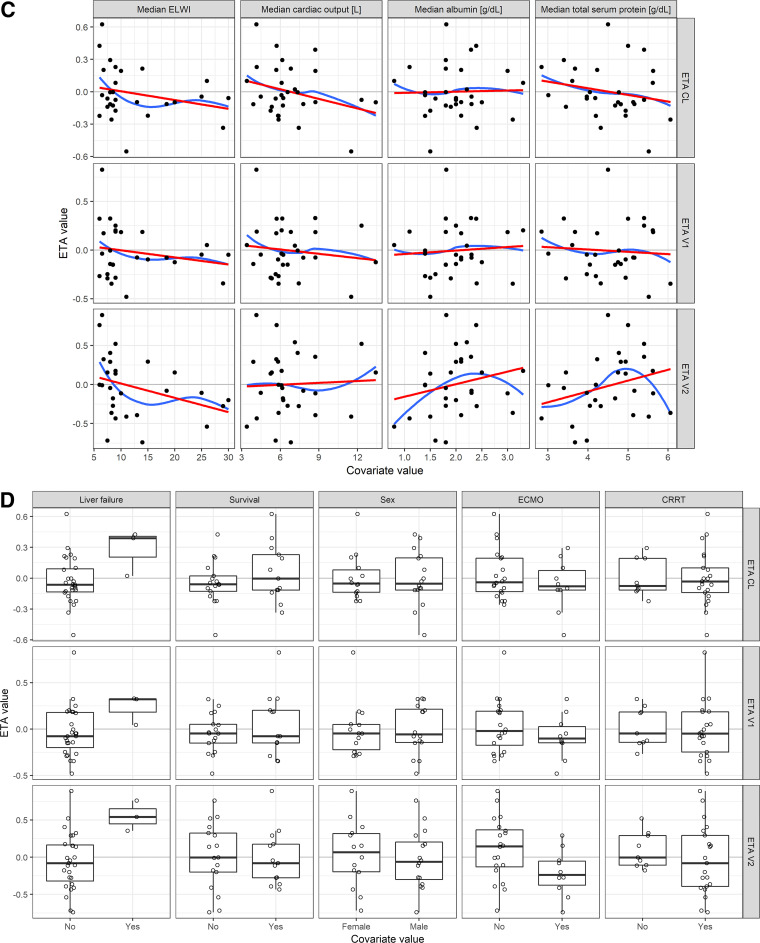
(A) Individual estimates for ETA (deviation of the individual estimate from the population mean) in relation to time-independent continuous covariates. The lines indicate trends in the data (red, linear function; blue, loess smooth). (B) Individual estimates for ETA in relation to median values of time-dependent continuous covariates: SOFA score, PCT concentration, dialysis dose, and ultrafiltration rate. The lines indicate trends in the data (red, linear function; blue, loess smooth). (C) Individual estimates for ETA in relation to median values of time-dependent continuous covariates: elevated lung water index, cardiac output albumin concentration, and total serum protein concentration. The lines indicate trends in the data (red, linear function; blue, loess smooth). (D) Individual estimates for ETA in relation to categorical covariates.

**FIG 6:**
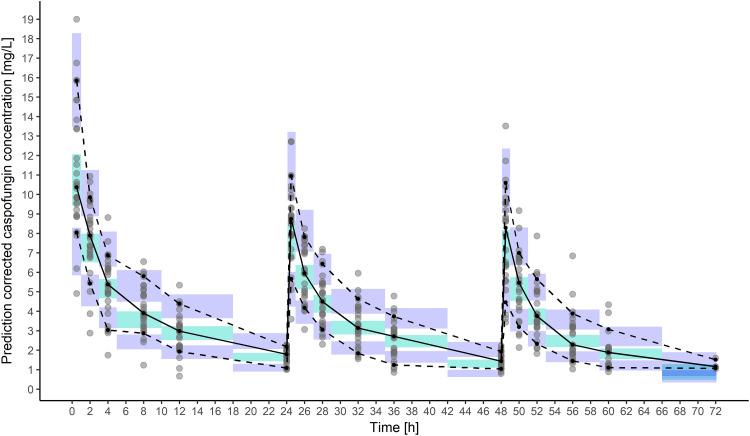
Prediction-corrected visual predictive check (VPC) showing the simulation-based 90% confidence intervals around the 10th, 50th, and 90th percentiles of the pharmacokinetic data in the form of turquoise (50th) and violet (10th and 90th) areas. The corresponding percentiles from the observed data are plotted in black.

The IIV model was significantly improved by including unique typical values for CL and *V*_1_ on each occasion (a decrease in objective function value by 145.82). The model with three separate CL and *V*_1_ values was further tested for covariate effects of time-independent covariates, the median values of time-dependent covariates, and categorical covariates. These relationships are shown in Fig. 5. Variables showing signs of correlation were formally tested in the model. No significant covariate effect was found. The final model parameter summary is shown in Table 2, and the predictive model performance is presented in Fig. 6.

**TABLE 2:** Summary of final model parameters^*a*^

Parameter	Estimate (%RSE)	Shrinkage (%)	Bootstrap median (90% CI)
θ_CL_ day 1 (liter/h)	0.563 (6.7)		0.558 (0.495–0.618)
θ_CL_ day 2 (liter/h)	0.737 (6.1)		0.734 (0.668–0.817)
θ_CL_ day 3 (liter/h)	1.01 (9.1)		1.00 (0.854–1.17)
θ*_V_*_1_ day 1(liter)	6.04 (7.0)		6.02 (5.39–6.75)
θ*_V_*_1_ day 2 (liter)	7.32 (5.3)		7.28 (6.56–8.09)
θ*_V_*_1_ day 3 (liter)	7.70 (5.3)		7.64 (6.83–8.62)
θ*_Q_* (liter/h)	1.31 (15.7)		1.31 (1.03–1.76)
θ*_V_*_2_ (liter)	5.13 (13.7)		5.18 (4.04–6.49)
			
Interindividual variability			
ω^2^_CL_ (%CV)	24.7 (27.3)	1.7	0.243 (0.184–0.296)
ω^2^*_V_*_1_ (%CV)	28.2 (39.6)	4.1	0.274 (0.182–0.356)
ω^2^*_Q_* (%CV)	0 FIX		
ω^2^*_V_*_2_ (%CV)	49.4 (37.1)	17.4	0.502 (0.327–0.690)
cor_CL-_*_V_*_1_	0.868 (34.9)		0.886 (0.611–1.00)
			
Residual error model			
σ^2^_prop_ (%CV)	19.9 (4.4)		0.195 (0.181–0.209)

a Terms: σ^2^_prop_, variance of proportional residual random error; RSE, relative standard error; %CV, percent coefficient of variation; CL, clearance; *Q*, intercompartmental clearance; *V*_1_, volume of distribution of the central compartment; *V*_2_, volume of distribution of the peripheral compartment; cor, correlation; 90% CI, 90% confidence interval of the parameter estimate derived from a nonparametric bootstrap analysis (*n* = 1,000).

**FIG 7:**
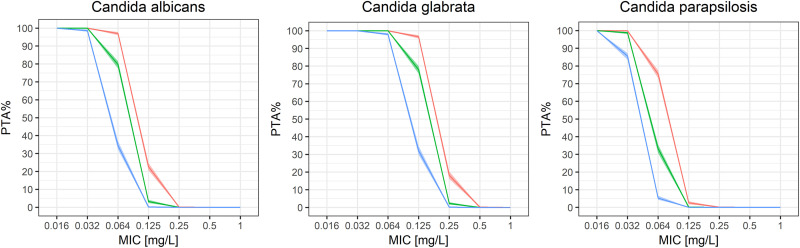
Probabilities of target attainment (PTA) of caspofungin based on preclinical targets for C. parapsilosis, C. glabrata, and C. albicans for the three clearance values estimated for three subsequent dosing occasions (first occasion in red, second occasion in green, and third occasion in blue) with colored area around the lines representing 90% confidence intervals based on bootstrap samples.

Based on the estimated model parameters, probability of target attainment (PTA) based on preclinical targets for Candida species (14) was calculated for the first, second and third day of caspofungin therapy (Table 3 and Fig. 7).

**TABLE 3:** Probability of target attainment of caspofungin for C. albicans, C. glabrata, and C. parapsilosis by MIC values based on preclinical targets and clearance values estimated for 3 days of therapy with confidence intervals based on bootstrap samples^*a*^

Organism and PTA variable	PTA data determined at various caspofungin MICs and times after the first dose of caspofungin																				
	0.016 mg/liter			0.032 mg/liter			0.064 mg/liter			0.125 mg/liter			0.25 mg/liter			0.5 mg/liter			1 mg/liter		
	Day 1	Day 2	Day 3	Day 1	Day 2	Day 3	Day 1	Day 2	Day 3	Day 1	Day 2	Day 3	Day 1	Day 2	Day 3	Day 1	Day 2	Day 3	Day 1	Day 2	Day 3
C. albicans																					
Median	100.0	100.0	100.0	100.0	100.0	98.7	97.0	79.6	34.4	22.7	3.2	0.2	0.1	0.0	0.0	0.0	0.0	0.0	0.0	0.0	0.0
5th percentile	100.0	100.0	100.0	100.0	99.8	98.0	96.1	77.6	32.4	20.6	2.3	0.0	0.1	0.0	0.0	0.0	0.0	0.0	0.0	0.0	0.0
95th percentile	100.0	100.0	100.0	100.0	100.0	99.2	97.9	81.6	36.9	24.8	4.1	0.5	0.3	0.0	0.0	0.1	0.0	0.0	0.0	0.0	0.0
																					
C. glabrata																					
Median	100.0	100.0	100.0	100.0	100.0	100.0	100.0	99.9	98.0	96.6	77.7	32.1	18.2	2.2	0.1	0.1	0.0	0.0	0.0	0.0	0.0
5th percentile	100.0	100.0	100.0	100.0	100.0	100.0	100.0	99.8	97.3	95.6	75.6	30.1	16.4	1.5	0.0	0.1	0.0	0.0	0.0	0.0	0.0
95th percentile	100.0	100.0	100.0	100.0	100.0	100.0	100.0	100.0	98.8	97.5	79.9	34.5	20.2	3.0	0.4	0.2	0.0	0.0	0.1	0.0	0.0
																					
C. parapsilosis																					
Median	100.0	100.0	100.0	99.9	98.7	85.6	75.4	33.1	5.3	2.5	0.1	0.0	0.1	0.0	0.0	0.0	0.0	0.0	0.0	0.0	0.0
5th percentile	100.0	100.0	99.9	99.8	98.1	83.8	73.2	30.8	4.2	1.7	0.0	0.0	0.0	0.0	0.0	0.0	0.0	0.0	0.0	0.0	0.0
95th percentile	100.0	100.0	100.0	100.0	99.3	87.4	77.6	35.6	6.6	3.4	0.3	0.0	0.1	0.0	0.0	0.0	0.0	0.0	0.0	0.0	0.0

a PTA, probability of target attainment.

## DISCUSSION

The phenomenon of change in clearance and volume of the central compartment with time was not reported in the literature for caspofungin, including studies in ICU patients.

The literature studies report mean clearance values of 0.55 liters/h (7% relative standard error [7%RSE]) in ICU patients (7), 0.403 liters/h (5%RSE) in patients with invasive aspergillosis (value for a 76-kg patient) (15), 0.62 liters/h (19.02%RSE) in patients undergoing CRRT (16), 0.57 liters/h (interquartile range [IQR], 0.54 to 0.77) and 0.54 liters/h (IQR, 0.44 to 0.60 liters/h) on days 3 and 7 of the study in ICU patients (17), respectively, 0.66 liters/h (range, 0.37 to 1.26 liters/h) in ICU patients (4). Two studies in healthy subjects report CL values between 0.591 and 0.746 liters/h after a single dose (18), 0.631 liters/h (sampling for 28 days), and 0.579 liters/h (sampling for 26 weeks) (19).

Our three typical CL values of 0.563 liters/h (6.7%RSE), 0.737 liters/h (6.1%RSE), 1.01 liters/h (9.1%RSE) after the first, second, and third doses, respectively, were estimated with precision similar to other studies. Although the CL after the first dose (0.563 liters/h, 6.7%RSE) is well within the values reported in literature, the CL after the second dose (0.737 liters/h, 6.1%RSE) is within the highest previously reported values, and the CL after the third dose (1.01 liters/h, 9.1%RSE) almost doubles compared to CL after the first dose and literature CL values. Interestingly, the mean CL for all three occasions estimated during model building process was 0.709 liters/h, which is in agreement with the value provided in the drug label (0.72 liters/h) (6).

The study by Stone et al. (19) revealed slow excretion and biotransformation of caspofungin (peak of radioactivity excretion at 6 to 7 days) and indicates that the CL of caspofungin estimated using the pharmacokinetic data collected within the first 24 h to 30 h after dose administration might be biased due to confounding with the distribution and redistribution processes.

The shift in mean *V*_1_ values for the three dosing occasions in our model was also upward: 6.04 liters (7.0%RSE) after the first dose, 7.32 liters (5.3%RSE) after the second dose, and 7.70 liters (5.3%RSE) after the third dose.

The changes in parameters with time require further investigation as they might be caused by a process that our study was not able to capture and describe. It could be the result of temporal changes in protein binding as caspofungin is highly protein-bound (97%) (6). Another possibility is nonlinearity in metabolic or distribution processes.

Based on the estimated typical values of clearance after the first (CL1), second (CL2), and third (CL3) doses, the maintenance dose of 50 mg would result in typical AUC_0–24_ values of 89, 68, and 50 mg ⋅ h/liter, respectively. Following the rationale of van der Elst et al. (4) for determining sufficient caspofungin exposure, if we consider AUC_0–24_ value of 98 mg ⋅ h/liter as the minimal value required for efficacy, this target would not be achieved in any case, and the decreases in exposure between doses would be 24% (CL2 versus CL1) and 44% (CL3 versus CL1).

The distribution process might be inadequately captured by the proposed two-compartment model due to too-short sampling, which prevented fitting a more complicated model. The study by Stone et al. (19) described a long terminal phase of radioactive labeled caspofungin with a half-life of 14.6 days, with quantifiable concentrations as long as 22.3 weeks after administration. Since our sampling was only as late as 24 h after dose administration, the parameters of three-compartment model fitted to our data could not be estimated. Nevertheless, the nearly perfect calibration of the proposed model with the data (Fig. 6) allows making unbiased concentration predictions for similar patients and therapy durations shorter than 72 h.

In our model, a covariance term was estimated for CL-*V*_1_ interaction. This term was also included in the model proposed by Würthwein et al. (15), but the physiological background of this term was not further investigated. Since the route of administration of caspofungin is intravenous (i.v.), the interaction is not related to bioavailability, and the correlation between CL and *V*_1_ might be caused by their codependence on a covariate that was not monitored in this study (e.g., protein binding).

During covariate analysis, body weight and age were tested as potential covariates since they were reported as covariates in other works, but they did not improve the model fit. All suspected covariate effects were tested in the model, but none of them was helpful in explaining the variability in pharmacokinetic data. Since drug exposure is related to CL values, covariates affecting this parameter are crucial for determining dosing that would result in uniform exposures in all patients. However, the interindividual variability in CL estimated for our data was 24.7%, which makes it very difficult to find a covariate that would decrease this value to a clinically relevant extent (by >20%).

The dose level of caspofungin was reduced in patients with liver failure. Unfortunately, we did not have data on biochemical parameters that would describe and distinguish the patients. There were only three patients in whom the dose was reduced, so the formal assessment of this variable was difficult, though the data suggest that their individual parameters might be altered. Clearance estimated by Martial et al. (7) in ICU patients with Child-Pugh B score was 0.55 liters/h, while individual clearance values estimated for our patients with liver failure ranged from 0.58 liters/h up to 1.54 liters/h. Based on individual parameters estimated for these patients, their AUC_0–24_ with a maintenance dose of 35 mg is very low, with a maximum value of 61 mg ⋅ h/liter, which is only 62% of the value required for efficacy, and a minimal value going as low as 23 mg ⋅ h/liter, which constitutes 23% of the 98 mg ⋅ h/liter target. The target AUC_0–24_ would not be achieved even with a standard 50-mg maintenance dose, while with a higher maintenance dose of 70 mg this target would be achieved only with clearance values estimated for the first dose. The impact of liver failure on individual pharmacokinetic parameters should therefore be investigated in further studies.

The simulations of probability of target attainment on the first, second, and third days of the caspofungin therapy with maintenance doses of 50 mg (Table 3 and Fig. 7) show that on day 1 in invasive candidiasis with an MIC of ≤0.125 mg/liter the PTA values were 22.6, 96.6, and 2.5% for C. albicans, C. glabrata, and C. parapsilosis, respectively; however, on day 3 these values were significantly lower, rendering therapeutic failure and the possible emergence of caspofungin resistance. The problem of target attainment with a standard 50-mg dose of caspofungin was also addressed in the study by Perez-Pitarch et al. (5). According to these authors, MIC values of ≤0.1 mg/liter resulted in achieving the PTA values of 54.1% for C. albicans and 99.9% for C. glabrata, but only 16.8% for C. parapsilosis, when a standard dose of caspofungin was used. We hypothesize that the discrepancy between our study and the study by Perez-Pitarch et al. (5) might arise from the differences between analyzed populations, e.g., higher score in a SOFA (sequential organ failure assessment) scale and a large proportion of extracorporeal therapies in our cohort.

The major limitation of our study is the premature termination of sample collection, which makes it impossible to determine whether the observed fall in caspofungin concentrations is halted on day 3, and the steady state is reached. This was mainly due to the fact that all the previously published studies on caspofungin pharmacokinetics assumed that the steady state is achieved on day 3; therefore, the study protocol was chosen accordingly (e.g., studies by Perez-Pitarch et al. [5], Muilwijk et al. [17], and Aguilar et al. [20]). Another significant limitation of our study is the lack of adequate monitoring of the efficacy of the antifungal treatment due to the unavailability of 1,3-β-d-glucan test in Poland, which is recommended by the recent guidelines.

### Conclusions.

The pharmacokinetics of caspofungin in our study was nonstationary since clearance and volume of the central compartment were changing over 72-h period of the study. The change in parameters with time was not explained by any of the recorded covariates. Increasing clearance with subsequent doses was associated with a clinically relevant decrease in caspofungin exposure (>20%). The use of ECMO, CRRT, albumin concentration, and other covariates did not significantly affect caspofungin pharmacokinetics. However, it is possible that the nonstationarity of caspofungin pharmacokinetics is characteristic for the studied group of patients (with sepsis pathophysiology together with invasive treatment such as ECMO or CRRT). Additional pharmacokinetic studies are urgently required to assess the possible lack of acquiring steady-state and suboptimal concentrations of the drug.

## MATERIALS AND METHODS

### Patients and study design.

This study was registered at ClinicalTrials.gov under ID NCT03399032. It was a prospective observational study conducted in adult critically ill patients admitted to ICU due to severe sepsis requiring broad-spectrum antibiotics with a high risk of fungal infection. Both medical and surgical patients with any type of infection (pulmonary, abdominal, urinary tract, etc.) were included. Eligible consent was obtained from each patient or his or her attendant. Allergy to caspofungin, lack of consent to participate in the study, and age below 18 or above 80 years were the exclusion criteria. Thirty-three patients were enrolled in the study. Data from three patients were excluded from analysis due to technical issue with handling the samples, which made them unsuitable for analysis and the concentrations could not be determined.

The antibiotic regimen included broad-spectrum antibacterial antibiotics plus caspofungin as empirical antifungal therapy (70 mg i.v. on the first day, and 50 mg i.v. on the consecutive days) once daily. In three patients with liver failure caspofungin doses were reduced (50 mg i.v. initial and 35 mg i.v. maintenance dose). Any site of infection was treated in the manner.

Each patient’s hemodynamic parameters were recorded with the use of transpulmonary thermodilution technique (PICCO). Other therapies, i.e., ventilatory support, sedation, and antifungal agent, were given as required. Recorded patient characteristics included sex, age, weight, height, SOFA score, procalcitonin (PCT) concentration, dialysis dose, ultrafiltration rate (UF), extravascular water index (ELWI), cardiac output, albumin, and total plasma protein concentrations.

Blood samples (3 ml) were collected 0.5, 2, 4, 8, 12, and 24 h after each dose of caspofungin for three consecutive days from the standard arterial cannula. Thirty minutes after each sample collection, blood was centrifuged for 10 min at 3,000 rpm. Subsequently, the supernatant was collected and frozen. Serum caspofungin concentrations were measured with high-performance liquid chromatography (HPLC).

### Assay.

Plasma samples were prepared for analysis according to a previously described procedure (3). A Dionex chromatographic system (Dionex, Sunnyvale, CA), equipped with a P580 LPG LC-6A gradient pump, UVD340S UV-visible detector, and a Rheodyne 7725 loop injector with a sufficient volume of 20 μl was employed in this study. Chromatographic separation was achieved on a Zorbax SB C_18_ column (150 × 4.6 mm × 5 μm) in isocratic mode. The mobile phase consisted of 20 mM triethylammonium phosphate buffer (pH 3.0): acetonitrile-methanol (50:40:10 [vol/vol/vol]; Fluka, HPLC grade). The mobile phase flow rate was 1.0 ml/min, and caspofungin absorbance was measured at 210 nm. The analytical method was validated in terms of linearity, the limits of detection (LOD) and quantification (LOQ), precision, and accuracy. The standard curves were linear over a concentration range from 0.25 to 10.0 μg/ml with an *R*^2^ of 0.987. The LOD and LOQ values for caspofungin were 0.08 and 0.18 μg/ml, respectively. The intra- and interday accuracies ranged from 98.1 to 102.8%, and the coefficients of variation (CVs) were between 0.8 and 3.4%.

### Pharmacokinetic modeling.

Population nonlinear mixed-effects modeling was conducted using NONMEM software (v7.3; Icon Development Solutions, Ellicott City, MD), GNU Fortran 95 compiler (GCC 4.6.0), and Wings for NONMEM (WFN741, http://wfn.sourceforge.net). The first-order conditional estimation method with η-ε interaction was employed throughout the model-building procedure. R computing environment (R Core Team 2019) was used for data processing and visualization.

The minimum value of the NONMEM objective function (OFV), typical goodness-of-fit diagnostic plots, and the evaluation of the precision of pharmacokinetic parameters and variability estimates were used to discriminate between various models during the model-building process. For hierarchical models, the decrease in OFV of 6.62 units defining an improved fit at a *P* of <0.01 for one additional parameter was considered to be statistically significant. The uncertainty of final model parameters was determined by nonparametric bootstrap with 1,000 replicates. The 90% confidence intervals (90% CI) for model parameters were constructed based on bootstrap results.

The model predictive performance was assessed by means of visual predictive check (VPC) based on 1,000 data sets generated from the parameters and variances of the final model. Prediction correction of concentrations was implemented due to two dosing regimens employed in the study. The observed and simulated concentrations were binned across time. The estimated 90% confidence intervals around the median and the 10th and 90th percentiles of the simulated concentrations were plotted against time with the median and 10th and 90th percentiles of the observed concentrations.

During the structural model-building step, candidate one-, two-, and three-compartment models were tested to describe the pharmacokinetics of caspofungin. The interindividual variability of the pharmacokinetic parameters was modeled in terms of η variables and was assumed to have a log-normal distribution with mean 0 and variance ω^2^. The interindividual random effects were examined graphically in terms of their distribution and correlation and the most correlated effects were tested in the model. Interoccasion variability of the pharmacokinetic parameters was modeled in terms of κ variables on three occasions (three subsequent doses) and was assumed to have log-normal distribution with mean 0 and variance π^2^.

The residual error for observations was modeled using the proportional error model with ε variable representing the proportional residual variability. It was assumed that *ε* is normally distributed with the mean 0 and variance σ^2^.

### Covariance analysis.

Covariates recorded in each patient included time-independent variables (age, weight, and height), time-dependent variables (SOFA score, PCT concentration, dialysis dose, UF, ELWI, cardiac output, albumin, and total plasma protein concentrations), and categorical variables (sex, survival, the use of ECMO and CRRT, and liver failure). All covariates were graphically explored to check for correlation. Preliminary screening of covariates was performed by visual inspection. Covariates that showed relationship with individual pharmacokinetic parameters were formally tested in the model.

The effect of continuous covariates on pharmacokinetic parameters was tested as a linear relationship. The allometric relationship was also tested to adjust volume and clearance parameters for body weight, centered around the weight of a typical 70-kg patient:

(1) $$P=\theta_{P}\cdot {\left(\frac{\mathrm{BW}}{70}\right)}^{a}$$

with the exponent *a* equal to 1 for volume terms and 0.75 for clearance terms.

For categorical covariates, each parameter was tested for the difference between two groups using indicator variable:

(2) $$P=\theta_{1}+\theta_{2}\cdot \mathrm{IND}$$

where *θ_1_* is the typical parameter value for one group of patients and θ_2_ is the difference in typical parameter value between two groups.

### Simulations of probability of target attainment.

The simulations of PTA were based on model parameters from bootstrap samples (*n* = 1,000) in order to include the parameter uncertainty. Predictions of AUC at steady state were performed for the standard 50-mg maintenance dose. The target AUC/MIC values are based on preclinical pharmacodynamic targets from neutropenic murine disseminated candidiasis model for C. albicans (AUC/MIC = 865), C. glabrata (AUC/MIC = 450), and C. parapsilosis (AUC/MIC = 1,185) (18). PTA was obtained by counting subjects who achieved the target AUC/MIC for MIC values from 0.125 to 2 mg/liter.
